# Supplementary material for: Sustained-Release Powders Based on Polymer Particles for Pulmonary Delivery of Beclomethasone Dipropionate in the Treatment of Lung Inflammation
Source: Pharmaceutics. 2023 Apr 14;15(4):1248. doi: 10.3390/pharmaceutics15041248 (PMC10144675; doi:10.3390/pharmaceutics15041248)
Supplement: Supplementary file 1 [file pharmaceutics-15-01248-s001.zip › pharmaceutics-2285989-supplementary.pdf]

Article

Supplementary materials: Sustained-release powders based on polymer particles for pulmonary delivery of beclomethasone dipropionate in the treatment of lung inflammation

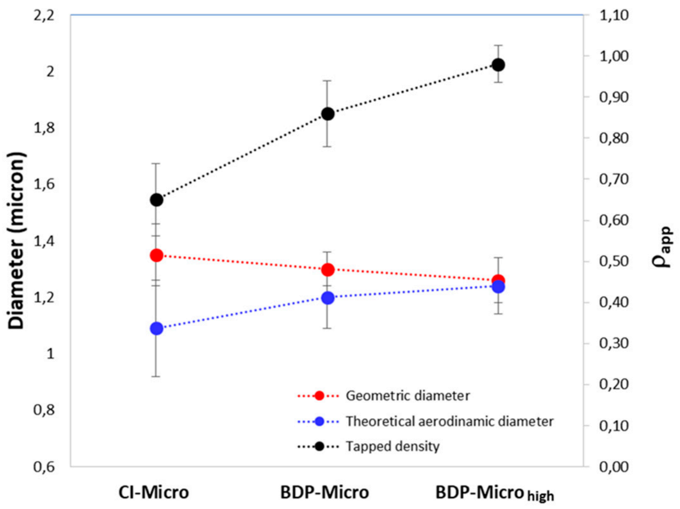

Figure S1. Values of tapped density (δ_{tapp}), mean geometric diameter (d_g), theoretical aerodynamic diameter (d_{aer}) of samples CI-Micro, BDP-Micro$_{high}$, and BDP-Micro.

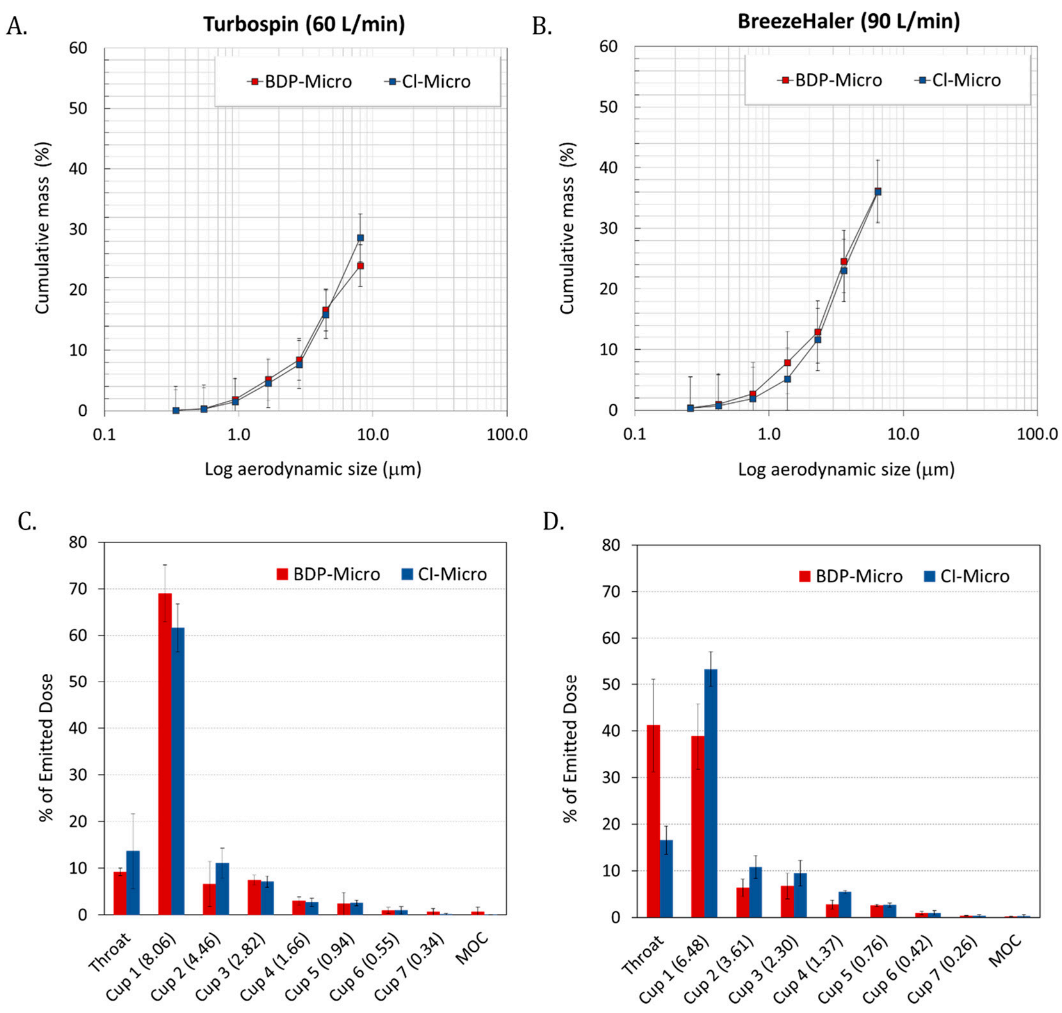

Figure S2. In vitro aerosol performance of BDP-Micro and Cl-Micro upon delivery through Next Generation Impactor (NGI). The formulations were delivered the medium-resistance DPI Turbospin® (PH&T Pharma, Italy) and the low-resistance DPI Breezhaler® (RS01, Plastiape, Italy) at 60 and 90 L/min, respectively. (**A**,**B**) Cumulative mass recovered as a function of the cut-off diameter of the NGI; (**C**,**D**) NGI deposition pattern. Data are the mean ± SD of values calculated on three different batches.
